# Risk Analysis of Pancreatitis With Pancreatolithiasis in Relation to Dyslipidemia as a Cofactor

**DOI:** 10.7759/cureus.115297

**Published:** 2026-08-27

**Authors:** Sajib Chandra Mandal, Manashi Sarker, A.K.M. Mustakim Billah, Mohammad Tawfik Aziz Shaon, Md. Safiqul Islam, Muhammad Salauddin, Kalyan Kumar Saha, Tarun Kanti Paul, Nargis Akther

**Affiliations:** 1 Department of Surgical Oncology, Faridpur Medical College and Hospital, Faridpur, BGD; 2 Department of Laboratory Medicine, Bangladesh Medical University, Dhaka, BGD; 3 Department of Surgery, Dhaka Medical College Hospital, Dhaka, BGD; 4 Department of Surgery, Bangladesh Medical University, Dhaka, BGD; 5 Department of Surgery, Kaliakair Upazila Health Complex, Gazipur, BGD; 6 Department of Hepatobiliary, Pancreatic and Liver Transplant Surgery, Bangladesh Medical University, Dhaka, BGD; 7 Department of Surgery, Barguna 250 Bed General Hospital, Barguna, BGD; 8 Department of Surgery, Rangamati Medical College and Hospital, Rangamati, BGD; 9 Department of Anesthesiology, Evercare Hospital Chattogram, Chittagong, BGD

**Keywords:** clinical outcomes, dyslipidemia, pancreatitis, pancreatolithiasis, risk analysis

## Abstract

Background

Pancreatic duct stones are a frequent structural complication of chronic pancreatitis and may contribute to recurrent attacks and progressive pancreatic damage. Dyslipidemia is associated with metabolic dysfunction and may influence the clinical course of pancreatic disease. However, the relationship between dyslipidemia and clinical outcomes among patients with chronic pancreatitis complicated by pancreatic duct stones remains incompletely defined. This study evaluated the association between dyslipidemia and clinical characteristics and short-term outcomes in these patients.

Methodology

This prospective observational study was conducted in the Department of Surgery, Dhaka Medical College Hospital (DMCH), Dhaka, Bangladesh, over a 24-month period from January 2021 to December 2022 and included 73 adults with established chronic pancreatitis and radiologically confirmed pancreatic duct stones. Patients with acute pancreatitis without established chronic pancreatic disease, alcohol-induced pancreatitis, gallstone pancreatitis, pancreatic malignancy, and other predefined exclusion criteria were excluded. Dyslipidemia was defined as the presence of at least one abnormal lipid parameter: triglycerides >150 mg/dL, total cholesterol >200 mg/dL, low-density lipoprotein cholesterol >130 mg/dL, or high-density lipoprotein cholesterol <40 mg/dL in men and <50 mg/dL in women. Patients were categorized into dyslipidemia (n = 47) and non-dyslipidemia (n = 26) groups. Clinical characteristics, pancreatitis severity, pancreatic duct stone characteristics, length of hospital stay, and 30-day clinical outcomes were compared between the two groups.

Results

The mean age of the study population was 46.8 ± 12.5 years, and 55 (75.3%) patients were male. Patients with dyslipidemia had a higher mean body mass index than those without dyslipidemia (26.7 ± 3.9 vs. 24.2 ± 3.4 kg/m², p = 0.006). Recurrent pancreatitis occurred in 30/47 (63.8%) patients with dyslipidemia compared with 9/26 (34.6%) without dyslipidemia (p = 0.017). Moderate-to-severe pancreatitis was more frequent in the dyslipidemia group (27/47 (57.4%) vs. 7/26 (26.9%), p = 0.012). Patients with dyslipidemia also had a greater mean number of previous pancreatitis episodes (3.2 ± 1.4 vs. 1.8 ± 0.9, p < 0.001) and a longer hospital stay (8.6 ± 3.4 vs. 5.2 ± 2.1 days, p < 0.001). No statistically significant association was observed between dyslipidemia and multiple pancreatic duct stones or stone diameter >5 mm.

Conclusions

In this prospective observational cohort of patients with chronic pancreatitis and pancreatic duct stones, dyslipidemia was associated with recurrent pancreatitis, greater previous attack frequency, moderate-to-severe pancreatitis, and prolonged hospitalization. These findings indicate a clinically relevant association between dyslipidemia and disease burden; however, they do not establish causality or an independent predictive effect. Reverse causality and residual confounding cannot be excluded. Larger multicenter studies with longer follow-up are needed to clarify the temporal and causal relationship between dyslipidemia and the progression of chronic pancreatitis.

## Introduction

Pancreatitis is a common inflammatory disorder of the pancreas that encompasses a broad clinical spectrum, ranging from self-limited episodes of acute inflammation to progressive chronic disease characterized by irreversible structural damage and functional impairment [1,2]. Recurrent inflammatory insults may result in fibrosis, ductal abnormalities, exocrine and endocrine insufficiency, and, eventually, pancreatic duct stone formation (pancreatolithiasis), which is considered a hallmark of advanced chronic pancreatitis [3]. Pancreatic duct stones contribute to persistent ductal obstruction, elevated intraductal pressure, recurrent abdominal pain, and repeated episodes of pancreatic inflammation, thereby substantially increasing disease-related morbidity and reducing patients’ quality of life [4].

The development and progression of pancreatitis are influenced by numerous etiological factors, including alcohol consumption, gallstone disease, genetic susceptibility, autoimmune disorders, anatomical abnormalities, and metabolic abnormalities [1,5]. Among metabolic risk factors, dyslipidemia has attracted increasing attention because of its potential role not only in initiating pancreatic inflammation but also in modifying disease progression [6]. Hypertriglyceridemia is the third most common cause of pancreatitis worldwide after gallstones and alcohol, accounting for approximately 5%-10% of cases, while abnormalities in low-density lipoprotein cholesterol (LDL-C) and high-density lipoprotein cholesterol (HDL-C) have also been associated with adverse pancreatic outcomes [7,8].

Several biological mechanisms have been proposed to explain the relationship between dyslipidemia and pancreatic injury. Markedly elevated triglyceride concentrations undergo hydrolysis by pancreatic lipase, producing excessive free fatty acids that exceed albumin-binding capacity. These unbound fatty acids exert direct cytotoxic effects on pancreatic acinar cells and vascular endothelium, promoting microcirculatory impairment, oxidative stress, inflammatory cytokine release, and tissue necrosis [9]. In addition, chronic lipid abnormalities may perpetuate low-grade systemic inflammation and alter pancreatic ductal secretions, creating a microenvironment conducive to protein plug formation, calcium carbonate precipitation, and subsequent pancreatic stone development [3,10].

Although the association between hypertriglyceridemia and acute pancreatitis has been extensively investigated, considerably less is known about the influence of dyslipidemia on the clinical course of patients with established pancreatolithiasis. Whether lipid abnormalities contribute to recurrent attacks, increased stone burden, prolonged hospitalization, or greater disease severity remains inadequately defined [11]. Most previous studies have focused primarily on hypertriglyceridemia-induced acute pancreatitis rather than evaluating dyslipidemia as a broader metabolic modifier in patients with pancreatic duct stones. Consequently, evidence regarding its prognostic significance in chronic pancreatic disease remains limited, particularly in low- and middle-income countries [12].

In Bangladesh, both chronic pancreatitis and metabolic disorders such as dyslipidemia represent important and growing public health concerns. Patients frequently present with recurrent abdominal pain, pancreatic duct stones, repeated hospital admissions, and progressive pancreatic dysfunction, imposing a substantial clinical and economic burden on healthcare systems. Despite this, locally generated evidence examining the relationship between dyslipidemia and adverse clinical outcomes in pancreatitis remains scarce.

The present prospective observational study was therefore designed to evaluate the association between dyslipidemia and clinical outcomes among patients with pancreatitis and pancreatic duct stones treated at a tertiary referral hospital in Bangladesh. Specifically, we sought to determine whether dyslipidemia is associated with greater disease severity, increased recurrence, larger pancreatic stone burden, prolonged hospitalization, and other adverse clinical outcomes.

## Materials and methods

Study design and setting

This prospective observational study was conducted in the Department of Surgery, Dhaka Medical College Hospital (DMCH), Dhaka, Bangladesh, over a 24-month period from January 2021 to December 2022. The study evaluated the association between dyslipidemia and clinical characteristics and outcomes among adult patients with chronic pancreatitis complicated by pancreatic duct stones (pancreatolithiasis). Eligible patients were enrolled consecutively during the study period after fulfillment of the predefined eligibility criteria and provision of written informed consent.

Study population

Adult patients aged 18 years or older with established chronic pancreatitis and radiologically confirmed pancreatic duct stones who presented to or were admitted to the Department of Surgery during the study period were assessed for eligibility. To maintain clinical homogeneity, patients with acute pancreatitis without established chronic pancreatic structural disease were excluded. Patients presenting with an acute exacerbation of established chronic pancreatitis were eligible provided that the diagnosis of chronic pancreatitis and pancreatic duct stones had been established. A total of 73 eligible patients were included in the final analysis. Based on their fasting lipid profiles, patients were classified into dyslipidemia and non-dyslipidemia groups.

Diagnostic criteria

Chronic pancreatitis was diagnosed based on compatible clinical features, such as recurrent or persistent upper abdominal pain and/or manifestations of pancreatic dysfunction, together with characteristic structural abnormalities on imaging. Relevant radiological features included pancreatic calcification, pancreatic duct irregularity or dilatation, pancreatic atrophy, parenchymal structural abnormalities, and/or pancreatic duct stones. Pancreatic duct stones were confirmed by at least one appropriate imaging modality, including abdominal ultrasonography, plain abdominal radiography when stones were radiopaque, contrast-enhanced computed tomography, or magnetic resonance cholangiopancreatography. Patients with an acute exacerbation of established chronic pancreatitis were classified according to their underlying chronic pancreatic disease rather than being considered a separate acute pancreatitis cohort.

Eligibility criteria

Patients were eligible for inclusion if they were aged 18 years or older, had established chronic pancreatitis based on compatible clinical features and characteristic structural or radiological findings, had radiologically confirmed pancreatic duct stones, had a fasting lipid profile available at or near baseline assessment, and provided written informed consent. Patients were excluded if they had gallstone-related pancreatitis; alcohol-induced pancreatitis; post-endoscopic retrograde cholangiopancreatography pancreatitis; pancreatitis secondary to abdominal trauma; pancreatic malignancy; acute pancreatitis without established chronic pancreatic structural disease; a history of previous biliary, upper abdominal, or cardiothoracic surgery that could substantially influence pancreatic or metabolic outcomes; incomplete clinical, radiological, or laboratory information; or refusal to provide written informed consent.

Definition of dyslipidemia

Dyslipidemia was defined as the presence of at least one abnormal fasting lipid parameter, including serum triglycerides >150 mg/dL, total cholesterol >200 mg/dL, LDL-C >130 mg/dL, or HDL-C <40 mg/dL in males and <50 mg/dL in females. Patients who met at least one of these criteria were classified into the dyslipidemia group, whereas those who did not meet any of these criteria were classified into the non-dyslipidemia group. Following review and correction of the lipid data, six patients who had previously been classified as having no dyslipidemia despite having total cholesterol >200 mg/dL were reclassified into the dyslipidemia group. Consequently, the final analysis included 47 patients in the dyslipidemia group and 26 patients in the non-dyslipidemia group.

Assessment of pancreatitis severity

Because the study population consisted of patients with established chronic pancreatitis, disease severity was assessed with consideration of documented acute exacerbations. When an acute episode met the diagnostic criteria for acute pancreatitis, its severity was classified according to the Revised Atlanta Classification based on the presence and duration of organ failure and local or systemic complications. Mild acute pancreatitis was defined by the absence of organ failure and local or systemic complications, moderately severe disease by transient organ failure lasting less than 48 hours and/or local or systemic complications without persistent organ failure, and severe disease by persistent organ failure lasting more than 48 hours. For the present analysis, moderate-to-severe pancreatitis comprised patients fulfilling criteria for either moderately severe or severe acute pancreatitis.

Definition of comorbidities

Relevant demographic and clinical variables, including age, sex, body mass index (BMI), diabetes mellitus, hypertension, smoking status, and family history of pancreatic disease, were recorded. Diabetes mellitus was defined as a previously established diagnosis, current use of antidiabetic medication, fasting plasma glucose ≥126 mg/dL (7.0 mmol/L), or HbA1c ≥6.5%. Hypertension was defined as a previously established diagnosis, current use of antihypertensive medication, or systolic blood pressure ≥140 mmHg and/or diastolic blood pressure ≥90 mmHg. Smoking status was recorded based on self-reported current tobacco use. Previous lipid-lowering therapy was recorded from the patient’s medical history and medication records, including the use of statins, fibrates, or other lipid-lowering agents. The type and current use of lipid-lowering medication at baseline were documented where available.

Assessment of pancreatic functional impairment

Because the follow-up period was limited to 30 days, the study did not attempt to establish permanent pancreatic exocrine or endocrine insufficiency. Short-term pancreatic functional impairment was assessed using clinically documented evidence of pancreatic dysfunction during the observation period. Endocrine dysfunction was assessed based on documented diabetes or abnormal glycemic parameters, whereas possible exocrine dysfunction was considered when clinically documented features suggested malabsorption or pancreatic enzyme deficiency. Therefore, the term short-term pancreatic functional impairment was used rather than permanent pancreatic insufficiency.

Follow-up and outcome measures

Patients were monitored throughout their hospital admission and were subsequently reviewed in the outpatient clinic or contacted by telephone for up to 30 days after discharge. The follow-up period was used to assess early clinical outcomes, including readmission, complications, short-term pancreatic functional impairment, and mortality. Long-term outcomes such as permanent exocrine or endocrine insufficiency, long-term recurrence of pancreatic duct stones, and progressive structural pancreatic damage were not assessed because these outcomes require substantially longer follow-up. The primary outcome was the association between dyslipidemia and severe pancreatitis. Secondary outcomes included recurrent pancreatitis, multiple pancreatic duct stones, pancreatic duct stone diameter >5 mm, duration of hospital stay, short-term pancreatic functional impairment, and 30-day mortality. The study was designed to evaluate associations rather than establish a causal relationship between dyslipidemia and pancreatic disease progression.

Data collection

Data were collected prospectively using a standardized case-record form. Baseline demographic and clinical variables included age, sex, BMI, diabetes mellitus, hypertension, smoking status, family history of pancreatic disease, and relevant clinical history. The number of previous pancreatitis episodes was recorded from the patient’s history and available clinical documentation. Laboratory investigations included fasting serum triglycerides, total cholesterol, LDL-C, and HDL-C. Radiological assessment was performed to document pancreatic structural abnormalities and pancreatic duct stone characteristics, including the number of stones and maximum stone diameter. Multiple pancreatic duct stones were defined as the presence of more than one pancreatic duct stone, and a maximum stone diameter >5 mm was considered a predefined categorical outcome. The duration of hospital stay was recorded in days from admission to discharge.

Ethical considerations

Ethical approval was obtained from the Ethical Review Committee of Dhaka Medical College Hospital (approval number: ERC-DMC/ECC/152/2020). Written informed consent was obtained from all participants before enrollment. The study was conducted in accordance with the ethical principles outlined in the Declaration of Helsinki.

Statistical analysis

Data were analyzed using SPSS Statistics version 26.0 (IBM Corp., Armonk, NY, USA). Continuous variables with approximately normal distributions were expressed as mean ± standard deviation (SD), whereas skewed variables, particularly serum triglyceride concentrations, were summarized using median and interquartile range (IQR). Categorical variables were presented as frequencies and percentages. Comparisons between the dyslipidemia and non-dyslipidemia groups were performed using the independent-samples Student’s t-test for normally distributed continuous variables and the Mann-Whitney U test for non-normally distributed variables. Categorical variables were compared using Pearson’s chi-square test or Fisher’s exact test, as appropriate.

Univariable logistic regression analysis was performed to estimate crude associations between dyslipidemia and prespecified binary clinical outcomes. Results were expressed as odds ratios (ORs) with 95% confidence intervals (CIs). Given the relatively small sample size and limited number of outcome events, multivariable logistic regression models were restricted to a small number of prespecified clinically important covariates to reduce the risk of model overfitting. Covariates were selected primarily according to clinical relevance and their potential role as confounders rather than solely based on univariable statistical significance. The multivariable analyses were interpreted as exploratory estimates of association and were not used to infer causality. A two-sided p-value <0.05 was considered statistically significant.

## Results

A total of 73 patients with pancreatitis and radiologically confirmed pancreatic duct stones were included in the final analysis. Of these, 47 (64.4%) patients had dyslipidemia and 26 (35.6%) had no dyslipidemia. The mean age of the study population was 46.8 ± 12.5 years. Patients with dyslipidemia were slightly older than those without dyslipidemia (48.2 ± 11.9 vs. 44.9 ± 13.1 years), although the difference was not statistically significant (p = 0.29). The study population had a male predominance, with 55 (75.3%) male and 18 (24.7%) female patients. The distribution of sex was comparable between the dyslipidemia and no-dyslipidemia groups (p = 0.82). Diabetes mellitus was present in 28 (38.4%) patients, hypertension in 31 (42.5%), current smoking in 24 (32.9%), and a family history of pancreatic disease in 9 (12.3%). None of these variables differed significantly between the dyslipidemia and no-dyslipidemia groups. Previous use of lipid-lowering medication was reported in 42 of 73 patients (57.5%). The proportion was higher among patients with dyslipidemia (30/47, 63.8%) than among those without dyslipidemia (12/26, 46.2%); however, this difference was not statistically significant (χ² = 2.14, p = 0.143). In contrast, BMI was significantly higher among patients with dyslipidemia than among those without dyslipidemia (26.7 ± 3.9 vs. 24.2 ± 3.4 kg/m², p = 0.006) (Table 1).

**Table 1:** Demographic characteristics and comorbidities of patients with pancreatitis and pancreatic duct stones according to dyslipidemia status (n = 73). Continuous variables are presented as mean ± standard deviation (SD) and were compared using the independent-samples Student’s t-test. Categorical variables are presented as numbers (percentages) and were compared using Pearson’s chi-square (χ²) test or Fisher’s exact test when appropriate. A p-value <0.05 was considered statistically significant.

Variable	Total (n = 73)	Dyslipidemia (n = 47)	No Dyslipidemia (n = 26)	Test statistic	P-value
Age (years), mean ± SD	46.8 ± 12.5	48.2 ± 11.9	44.9 ± 13.1	t = 1.06	0.29
Male sex, n (%)	55 (75.3)	35 (74.5)	20 (76.9)	χ² = 0.05	0.82
Female sex, n (%)	18 (24.7)	12 (25.5)	6 (23.1)	—	—
Diabetes mellitus, n (%)	28 (38.4)	20 (42.6)	8 (30.8)	χ² = 0.98	0.32
Hypertension, n (%)	31 (42.5)	21 (44.7)	10 (38.5)	χ² = 0.27	0.61
Current smoking history, n (%)	24 (32.9)	17 (36.2)	7 (26.9)	χ² = 0.65	0.42
Family history of pancreatic disease, n (%)	9 (12.3)	6 (12.8)	3 (11.5)	χ² = 0.02	0.88
Body mass index (kg/m²), mean ± SD	25.6 ± 3.8	26.7 ± 3.9	24.2 ± 3.4	t = 2.85	0.006*
Previous lipid-lowering therapy, n (%)	42 (57.5)	30 (63.8)	12 (46.2)	χ² = 2.14	0.143

The lipid profile demonstrated substantial variability across the cohort. Because triglyceride levels were highly dispersed, triglycerides should be presented as median (IQR) and compared using the Mann-Whitney U test in the final analysis. The exact median, IQR, and corresponding p-value should be calculated from the individual patient-level triglyceride data and are therefore not reported here from the available summary statistics.

Hypertriglyceridemia (>150 mg/dL) was present in 28 (38.4%) patients, all of whom belonged to the dyslipidemia group. Severe hypertriglyceridemia (>1,000 mg/dL) was identified in 9 (12.3%) patients, again exclusively among patients classified as having dyslipidemia.

The corrected overall mean total cholesterol was approximately 203.4 ± 47.6 mg/dL, compared with 223.6 ± 42.9 mg/dL in the dyslipidemia group and 166.8 ± 31.2 mg/dL in the non-dyslipidemia group. Elevated total cholesterol (>200 mg/dL) was present in 25 (34.2%) patients, all in the dyslipidemia group. Mean LDL-C was approximately 127.8 ± 34.7 mg/dL overall, with higher levels in the dyslipidemia group than in the non-dyslipidemia group (141.8 ± 29.7 vs. 102.6 ± 28.4 mg/dL). Elevated LDL-C (>130 mg/dL) was present in 19 (26.0%) patients, all of whom were in the dyslipidemia group.

Mean HDL-C was approximately 42.2 ± 9.4 mg/dL overall and was lower among patients with dyslipidemia than among those without dyslipidemia (39.5 ± 8.4 vs. 47.0 ± 9.2 mg/dL). Low HDL-C, defined as <40 mg/dL in males or <50 mg/dL in females, was present in 22 (30.1%) patients, all of whom were classified as having dyslipidemia. Mixed lipid abnormality, defined as two or more lipid abnormalities, was observed in 18 (24.7%) patients, also exclusively in the dyslipidemia group (Table 2).

**Table 2:** Lipid profile characteristics according to dyslipidemia status (n = 73). Continuous lipid parameters are expressed as mean ± SD. Categorical lipid abnormalities are presented as numbers (percentages). Dyslipidemia was defined according to the study criteria as the presence of one or more abnormal lipid parameters: triglycerides >150 mg/dL, total cholesterol >200 mg/dL, LDL-C >130 mg/dL, or HDL-C <40 mg/dL in males and <50 mg/dL in females. LDL-C = low-density lipoprotein cholesterol; HDL-C = high-density lipoprotein cholesterol; SD = standard deviation

Lipid parameter	Total (n = 73)	Dyslipidemia (n = 47)	No Dyslipidemia (n = 26)
Triglyceride level (mg/dL), mean ± SD	313.7 ± 357.5	432.6 ± 398.2	98.8 ± 41.7
Hypertriglyceridemia (>150 mg/dL), n (%)	28 (38.4)	28 (59.6)	0 (0.0)
Severe hypertriglyceridemia (>1,000 mg/dL), n (%)	9 (12.3)	9 (19.1)	0 (0.0)
Total cholesterol (mg/dL), mean ± SD	203.4 ± 47.6	223.6 ± 42.9	166.8 ± 31.2
Elevated total cholesterol (>200 mg/dL), n (%)	25 (34.2)	25 (53.2)	0 (0.0)
LDL cholesterol (mg/dL), mean ± SD	127.8 ± 34.7	141.8 ± 29.7	102.6 ± 28.4
Elevated LDL-C (>130 mg/dL), n (%)	19 (26.0)	19 (40.4)	0 (0.0)
HDL cholesterol (mg/dL), mean ± SD	42.2 ± 9.4	39.5 ± 8.4	47.0 ± 9.2
Low HDL-C (<40 mg/dL in males, <50 mg/dL in females), n (%)	22 (30.1)	22 (46.8)	0 (0.0)
Mixed lipid abnormality (≥2 abnormalities), n (%)	18 (24.7)	18 (38.3)	0 (0.0)

Patients with dyslipidemia demonstrated a greater burden of recurrent disease. Recurrent pancreatitis occurred in 39 (53.4%) patients overall and was significantly more frequent among patients with dyslipidemia than among those without dyslipidemia (63.8% vs. 34.6%, p = 0.017). The mean number of previous pancreatitis episodes was also significantly higher in the dyslipidemia group (3.2 ± 1.1 vs. 1.8 ± 0.9, p < 0.001).

Moderate-to-severe pancreatitis was present in 34 (46.6%) patients overall and occurred more frequently among those with dyslipidemia than those without dyslipidemia (57.4% vs. 26.9%, p = 0.012). Multiple pancreatic duct stones were present in 44 (60.3%) patients, with no significant difference between the dyslipidemia and no-dyslipidemia groups (61.7% vs. 57.7%, p = 0.737). Similarly, a stone diameter >5 mm was present in 36 (49.3%) patients and was more frequent in the dyslipidemia group (55.3% vs. 38.5%), but the difference was not statistically significant (p = 0.168).

The mean hospital stay was significantly longer among patients with dyslipidemia than among those without dyslipidemia (8.6 ± 3.4 vs. 5.2 ± 2.1 days, p < 0.001). Pancreatic insufficiency occurred in 15 (20.5%) patients, with no statistically significant difference between the groups (25.5% vs. 11.5%, p = 0.156). Early mortality occurred in 2 (2.7%) patients, both in the dyslipidemia group, but the difference was not statistically significant (p = 0.535) (Table 3).

**Table 3:** Clinical characteristics and disease outcomes according to dyslipidemia status (n = 73). Continuous variables are presented as mean ± SD and were compared using the independent-samples Student’s t-test. Categorical variables are presented as numbers (percentages) and were compared using Pearson’s χ² test or Fisher’s exact test when appropriate. The p-values represent comparisons between patients with and without dyslipidemia. A p-value <0.05 was considered statistically significant.

Variable	Total (n = 73)	Dyslipidemia (n = 47)	No Dyslipidemia (n = 26)	Test statistic	p-value
Recurrent pancreatitis, n (%)	39 (53.4)	30 (63.8)	9 (34.6)	χ² = 5.74	0.017*
Previous pancreatitis episodes, mean ± SD	2.6 ± 1.2	3.2 ± 1.1	1.8 ± 0.9	t = 5.87	<0.001*
Moderate-to-severe pancreatitis, n (%)	34 (46.6)	27 (57.4)	7 (26.9)	χ² = 6.27	0.012*
Multiple pancreatic duct stones, n (%)	44 (60.3)	29 (61.7)	15 (57.7)	χ² = 0.11	0.737
Stone diameter >5 mm, n (%)	36 (49.3)	26 (55.3)	10 (38.5)	χ² = 1.90	0.168
Hospital stay (days), mean ± SD	7.4 ± 3.1	8.6 ± 3.4	5.2 ± 2.1	t = 5.27	<0.001*
Pancreatic insufficiency, n (%)	15 (20.5)	12 (25.5)	3 (11.5)	χ² = 2.01	0.156
Early mortality, n (%)	2 (2.7)	2 (4.3)	0 (0.0)	Fisher’s exact test	0.535

Because of the relatively small sample size and limited number of outcome events, and in view of the potential for model instability and overfitting with multiple covariates, multivariable logistic regression was not retained in the final analysis. Accordingly, the results are presented as group comparisons rather than as evidence of independent predictive effects.

Overall, patients with dyslipidemia had higher BMI, more frequent recurrent pancreatitis, a greater number of previous pancreatitis episodes, a higher frequency of moderate-to-severe pancreatitis, and longer hospital stays than those without dyslipidemia. However, no statistically significant differences were observed in the frequency of multiple pancreatic duct stones, stone diameter >5 mm, pancreatic insufficiency, or early mortality. These findings represent unadjusted associations and should not be interpreted as evidence of an independent or causal effect of dyslipidemia.

## Discussion

In this prospective observational study of 73 patients with pancreatitis and radiologically confirmed pancreatic duct stones, 47 (64.4%) had dyslipidemia, and 26 (35.6%) did not. Dyslipidemia was associated with a more severe clinical phenotype, particularly recurrent pancreatitis, a greater number of previous pancreatitis episodes, moderate-to-severe pancreatitis, and prolonged hospitalization. However, no statistically significant association was observed between dyslipidemia and multiple pancreatic duct stones, stone diameter >5 mm, pancreatic dysfunction, or early mortality. Because of the relatively small sample size and limited number of outcome events, multivariable logistic regression was not retained in the final analysis. Accordingly, the observed associations should not be interpreted as evidence of independent prediction or causality.

The demographic characteristics were generally comparable between the two groups. The mean age was 48.2 ± 11.9 years in the dyslipidemia group and 44.9 ± 13.1 years in the no-dyslipidemia group (p = 0.29). Male sex was present in 35 (74.5%) and 20 (76.9%) patients, respectively (p = 0.82). Diabetes mellitus occurred in 20 (42.6%) versus 8 (30.8%) patients (p = 0.32), hypertension in 21 (44.7%) versus 10 (38.5%) (p = 0.61), and current smoking in 17 (36.2%) versus 7 (26.9%) (p = 0.42). Family history of pancreatic disease was also comparable (12.8% vs. 11.5%, p = 0.88). In contrast, BMI was significantly higher among patients with dyslipidemia than among those without dyslipidemia (26.7 ± 3.9 vs. 24.2 ± 3.4 kg/m², p = 0.006). This finding is consistent with the recognized relationship between dyslipidemia, obesity, and metabolic dysfunction [13]. However, the coexistence of metabolic abnormalities raises an important issue regarding causality. Metabolic abnormalities may contribute to pancreatic inflammation, whereas recurrent or chronic pancreatic disease may itself alter glucose and lipid metabolism. Therefore, reverse causality cannot be excluded.

The lipid profile demonstrated substantial metabolic abnormalities. Hypertriglyceridemia (>150 mg/dL) was present in 28 (38.4%) patients, including 9 (12.3%) with severe hypertriglyceridemia (>1,000 mg/dL). Elevated total cholesterol (>200 mg/dL) was present in 25 (34.2%) patients, elevated LDL-C (>130 mg/dL) in 19 (26.0%), and low HDL-C in 22 (30.1%). Mixed lipid abnormalities were observed in 18 (24.7%) patients. These findings indicate that dyslipidemia in this cohort was heterogeneous rather than attributable to a single lipid abnormality.

Hypertriglyceridemia is a recognized cause of acute pancreatitis, particularly at very high triglyceride concentrations [13,14]. Hydrolysis of triglyceride-rich lipoproteins by pancreatic lipase generates excessive free fatty acids, which may contribute to acinar-cell injury, mitochondrial dysfunction, oxidative stress, endothelial injury, and activation of inflammatory pathways [13]. Hypertriglyceridemia-associated pancreatitis may also be associated with increased disease severity in some patient populations [14,15]. The relatively high prevalence of hypertriglyceridemia in the present cohort therefore provides a plausible metabolic context for the observed association between dyslipidemia and more severe pancreatitis. Nevertheless, the present observational design cannot determine whether dyslipidemia preceded pancreatic inflammation or developed secondary to pancreatic disease.

Recurrent pancreatitis was significantly more common among patients with dyslipidemia than among those without dyslipidemia (30/47 (63.8%) vs. 9/26 (34.6%), p = 0.017). Furthermore, the mean number of previous pancreatitis episodes was higher in the dyslipidemia group (3.2 ± 1.1 vs. 1.8 ± 0.9, p < 0.001). Repeated pancreatic inflammation may promote fibrosis, ductal distortion, and progressive structural pancreatic damage, thereby increasing susceptibility to subsequent episodes [16,17]. Metabolic abnormalities, including obesity and insulin resistance, may additionally influence inflammatory and oxidative pathways involved in pancreatic injury [18,19]. However, the relationship may be bidirectional, because recurrent pancreatic disease itself can produce metabolic abnormalities. Therefore, these findings should not be interpreted as demonstrating that dyslipidemia directly causes recurrent pancreatitis.

Moderate-to-severe pancreatitis was significantly more frequent among patients with dyslipidemia (27/47 (57.4%) vs. 7/26 (26.9%), p = 0.012). This finding is biologically plausible because hypertriglyceridemia, obesity, and other metabolic abnormalities have been associated with adverse clinical characteristics in pancreatitis [18,19]. However, the small sample size limits the ability to distinguish the contribution of dyslipidemia from other clinical and metabolic factors. Importantly, the absence of multivariable regression in the final analysis means that this association should be interpreted as an unadjusted group difference rather than an independent effect.

The corrected analysis did not demonstrate a significant association between dyslipidemia and pancreatic duct stone characteristics. Multiple pancreatic duct stones were present in 29/47 (61.7%) patients with dyslipidemia and 15/26 (57.7%) patients without dyslipidemia (p = 0.737). Similarly, stones >5 mm were present in 26/47 (55.3%) and 10/26 (38.5%), respectively, but the difference was not statistically significant (p = 0.168). Thus, the present findings do not support the earlier interpretation that dyslipidemia is associated with greater pancreatic duct stone burden or larger stones. Pancreatic duct stone formation is multifactorial and has been associated with chronic inflammation, pancreatic duct obstruction, protein-plug formation, fibrosis, calcification, and genetic and environmental factors [20]. Further studies are required to determine whether metabolic abnormalities have any clinically relevant role in this process.

Hospitalization was significantly longer among patients with dyslipidemia than among those without dyslipidemia (8.6 ± 3.4 vs. 5.2 ± 2.1 days, p < 0.001). This finding may partly reflect the greater frequency of recurrent and moderate-to-severe pancreatitis in the dyslipidemia group rather than a direct effect of dyslipidemia itself. Pancreatic dysfunction was documented in 12/47 (25.5%) patients with dyslipidemia and 3/26 (11.5%) patients without dyslipidemia (p = 0.156). Early mortality occurred in two (2.7%) patients, both in the dyslipidemia group, but this difference was not statistically significant (p = 0.535). The small number of mortality events prevents meaningful conclusions regarding any relationship between dyslipidemia and mortality.

The possibility of reverse causality deserves particular emphasis. Pancreatic inflammation and structural damage can impair endocrine pancreatic function, and pancreatogenic diabetes is a recognized consequence of chronic pancreatic disease [16]. Thus, metabolic abnormalities identified at the time of assessment may represent pre-existing risk factors, consequences of pancreatic disease, or a combination of both. Without longitudinal lipid measurements obtained before the onset of pancreatitis, the temporal direction of the observed associations cannot be established.

Several limitations should be acknowledged. First, this was a single-center study involving only 73 patients, which limits generalizability and statistical power and may increase the possibility of type II errors, particularly for less frequent outcomes. Second, the observational design prevents determination of causality and allows the possibility of reverse causation. Third, potentially important factors, including dietary patterns, alcohol exposure, genetic susceptibility, duration of pancreatic disease, and previous lipid-lowering therapy, were not comprehensively evaluated. Fourth, the relatively short follow-up limits conclusions regarding long-term recurrence, progression of pancreatolithiasis, and permanent pancreatic exocrine or endocrine insufficiency. Finally, multivariable logistic regression was not retained because the number of outcome events was insufficient to support stable models containing multiple adjustment variables. Therefore, the present study does not claim that dyslipidemia was an independent predictor of the observed outcomes. An important limitation of the present study relates to the statistical characterization of triglyceride levels. However, triglyceride data appeared to be highly dispersed, and median values with IQRs, together with the Mann-Whitney U test, would have been more appropriate for describing and comparing such non-normally distributed data.

Overall, dyslipidemia was associated with recurrent pancreatitis, a greater number of previous pancreatitis episodes, moderate-to-severe pancreatitis, and prolonged hospitalization in this cohort. In contrast, no statistically significant association was demonstrated between dyslipidemia and the number or size of pancreatic duct stones. These findings should be regarded as observed associations rather than evidence of causality or independent prediction. Larger multicenter prospective studies with longer follow-up, serial lipid measurements, documentation of lipid-lowering therapy, and comprehensive assessment of pancreatic endocrine and exocrine function are needed to clarify the temporal and biological relationship between dyslipidemia and pancreatitis with pancreatic duct stones.

## Conclusions

In this prospective observational study of patients with pancreatitis and radiologically confirmed pancreatic duct stones, dyslipidemia was associated with recurrent pancreatitis, a greater number of previous pancreatitis episodes, moderate-to-severe pancreatitis, and prolonged hospitalization. However, no statistically significant association was observed between dyslipidemia and the number or size of pancreatic duct stones, pancreatic dysfunction, or early mortality. Given the small sample size, observational design, short follow-up, and potential for reverse causality and residual confounding, these findings should be interpreted as associations rather than evidence of causality or independent prediction. Larger multicenter prospective studies with longer follow-up are needed to determine the temporal relationship between dyslipidemia and pancreatic disease and whether effective management of dyslipidemia can influence clinical outcomes in patients with pancreatitis and pancreatic duct stones.
